# The conundrum of a global tool for early childhood development to monitor SDG indicator 4.2.1

**DOI:** 10.1016/S2214-109X(21)00030-9

**Published:** 2021-02-25

**Authors:** Bolajoko O Olusanya, Mijna Hadders-Algra, Cecilia Breinbauer, Andrew N Williams, Charles R J Newton, Adrian C Davis

**Affiliations:** Centre for Healthy Start Initiative, Lagos, Nigeria; University of Groningen, University Medical Center Groningen, Division of Developmental Neurology, Department of Paediatrics, Groningen, Netherlands; Center for Healthy Development, Seattle, WA, USA; Virtual Academic Unit, Northampton General Hospital, Northampton, UK; KEMRI-Wellcome Trust Research Program, Centre for Geographic Medicine Research (Coast), Kilifi, Kenya; Population Health Science, London School of Economics, London, UK

The UN’s Sustainable Development Goals (SDGs) agenda, by contrast to the Millennium Development Goals, provides a global policy framework to address the quality of life of beneficiaries of the remarkable reduction in mortality in those younger than 5 years since the child survival revolution began in 1982.^1^ The SDGs explicitly commit all governments and the global health community to actions that will “ensure that all girls and boys have access to quality early childhood development, care and pre-primary education so that they are ready for primary education”.^1^ To monitor progress towards achieving this target, “the proportion of children under 5 years of age who are developmentally on track in health, learning and psychosocial well-being, by sex” was chosen as the sole indicator (SDG 4.2.1).^1^ The choice of this age group is consistent with other SDG targets for young children, including undernutrition, poverty, and mortality (panel).^2^ The extensive scientific literature on human brain development reflects the importance of early detection and intervention, especially for children with, or at risk of, developmental disabilities.^3^

However, the Inter-Agency and Expert Group on SDG Indicators (IAEG-SDGs), which was established in 2015 by the UN to facilitate the implementation of all indicators, has proposed the exclusion of all children younger than 24 months from SDG 4.2.1, subject to final approval by the UN Statistical Commission in March, 2021 (panel). This proposal is attributable to the inability of UNICEF, as the custodian agency, to provide a suitable population-level measure of functioning and development in children younger than 24 months. The Washington Group on Disability Statistics, which was mandated by the UN in 2001 to develop a composite measure of child functioning, has not yet succeeded in producing a tool that includes all children.^4^ Developing the required measure of child functioning for all children younger than 5 years is portrayed as very subjective, culturally dependent, and too complex.^4^ This perception has led to the introduction of the Early Childhood Development Index for children aged 24–59 months as the proxy measure for SDG 4.2.1.

The proposed exclusion of all children younger than 24 months from the sole global indicator for early childhood development is of great concern for several reasons. First, the proposal is incompatible with the existing goal (SDG 4) and target (SDG 4.2) that embrace all children (panel). Second, exclusion of the youngest children violates non-discrimination provisions of the UN Convention on the Rights of the Child and the Convention on the Rights of Persons with Disabilities, and contradicts the SDGs’ core principle of not leaving anyone behind.^1^ Third, children younger than 24 months are usually the most disadvantaged cohort and the greatest beneficiaries of early detection and intervention services. Their exclusion is likely to undermine sustainable political support and donor funding commitments towards early childhood development initiatives^5^ for the estimated 250 million children younger than 5 years at risk of not realising their developmental potential because of stunting and extreme poverty,^6^ and for more than 50 million children with developmental disabilities in low-income and middle-income countries.^7^ Fourth, the suggestion that creating population-level measures for children younger than 24 months is unachievable does not consider all the available survey tools (eg, the Global Scale for Early Development^8^ and Caregiver Reported Early Development Instruments^9^). Finally, the proposed exclusion will not facilitate the integrated approach to child survival, development, and wellbeing recommended by the WHO–UNICEF–*Lancet* Commission on child and adolescent health.^10^

Already, UNICEF is committed to giving priority in everything they do to “the most disadvantaged children and countries in greatest need”. Therefore, we have three recommendations. First, the proposed exclusion should not proceed. The IAEG-SDGs should fully disclose the current absence of a single measurable tool for early childhood development for all children younger than 5 years while rapidly harmonising existing survey tools towards a comprehensive Early Childhood Development Index instrument. Second, transparent and active engagements with all relevant stakeholders, particularly those from high-burden regions, should be prioritised. For example, UN conventions require people with disabilities, including children with disabilities, through their representative organisations, to be consulted and actively involved in the development and implementation of policies and programmes that directly affect them. To do otherwise will be unjust and improper. Finally, we should track the proportion of children with disabilities that receive intervention services as a key performance measure for early childhood development.

In conclusion, more than 5 years have passed since the SDGs were launched. Every day, the future of many child survivors is being hugely disrupted by life-long impairments. It is time to build trust and deliver services that will directly and positively impact the future wellbeing of all children early enough (<2 years of age) for the best possible developmental outcomes.

## *Panel:* Provisions for children younger than 5 years under the Sustainable Development Goals framework^2^

**Goal 2: end hunger, achieve food security, and improve nutrition and promote sustainable agriculture**

*Target 2.1*

By 2030, end hunger and ensure access by all people, in particular the poor and people in vulnerable situations, including infants, to safe, nutritious, and sufficient food all year round

*Indicators*

- 2.1.1: prevalence of undernourishment

*Target 2.2*

By 2030, end all forms of malnutrition, including achieving, by 2025, the internationally agreed targets on stunting and wasting in children younger than 5 years, and address the nutritional needs of adolescent girls, pregnant and lactating women, and older people

*Indicators*

- 2.2.1: prevalence of stunting (height for age more than two SDs lower than the median of WHO’s Child Growth Standards) among children younger than 5 years
- 2.2.2: prevalence of malnutrition (weight for height more than two SDs higher or lower than the median of WHO’s Child Growth Standards) among children younger than 5 years, by type (wasting and overweight)

**Goal 3: ensure healthy lives and promote wellbeing for all at all ages**

*Target 3.2*

By 2030, end preventable deaths of neonates and children younger than 5 years, with all countries aiming to reduce neonatal mortality to 12 deaths per 1000 livebirths or fewer, and mortality in children younger than 5 years to 25 deaths per 1000 livebirths or fewer

*Indicators*

- 3.2.1: under-5 mortality rate
- 3.2.2: neonatal mortality rate

***Goal* 4: ensure inclusive and equitable quality education and promote lifelong learning opportunities for all**

*Target 4.2*

By 2030, ensure that all girls and boys have access to quality early childhood development, care, and pre-primary education so that they are ready for primary education

*Indicators*

- 4.2.1: proportion of children younger than 5 years who are developmentally on track in health, learning, and psychosocial wellbeing, by sex

The portion of this indicator that measured progress for children aged 0–23 months was deleted in principle in March, 2020, subject to final approval by the UN Statistical Commission

- Revised 4.2.1 (2020): proportion of children aged 24–59 months who are developmentally on track in health, learning, and psychosocial wellbeing, by sex
